# Enhancing state epidemiology and laboratory capacity for infectious diseases.

**DOI:** 10.3201/eid0403.980331

**Published:** 1998

**Authors:** D A Deppe

**Affiliations:** Centers for Disease Control and Prevention, Atlanta, Georgia, USA.


### Enhancing State Epidemiology and Laboratory Capacity for Infectious Diseases

**Deborah A. Deppe**
Centers for Disease Control and Prevention, Atlanta, Georgia, USA

The Epidemiology and Laboratory Cooperative is an agreement that provides state and large local health departments with resources to strengthen and enhance basic capacity for public health surveillance and response for infectious diseases. The funding is used to implement new technology, upgrade systems, train staff, and purchase office and laboratory equipment. Six of the 30 sites reported on their programs.

The Vermont Department of Health has undertaken statewide efforts to improve communicable disease reporting through legislation. Before 1997, public health law required physicians, nurses, hospital administrators, and school and town health officials to report communicable disease (defined by regulation) to the Commissioner of Health. Legislation proposed and passed in 1997 required health maintenance organizations and managed care organizations to report as well. This model law defines such information as "confidential and privileged" and extends protection to investigations and studies of disease outbreaks.

The Kansas Department of Health and Environment is building epidemiology and laboratory capacity by expanding electronic surveillance and analysis, enhancing surveillance for diarrheal disease, and integrating information from other sources into the existing surveillance system. The new programs are flexible so they can meet the challenges posed by new and emerging infectious diseases as well as changing needs within public health.

The New York State Department of Health uses electronic communications to enhance rapid reporting of communicable disease epidemiologic and laboratory data within the state. In July 1997, seven counties began pilot testing the New York Health Information Network (HIN), a secure Intranet site. Web forms were designed for designated county personnel to submit confidential case and supplemental information on 61 reportable communicable diseases on the HIN. Since implementation, reports from local health departments to the state have been more timely. Counties can easily update and query their own data and can access statewide reports generated by the state and posted on the HIN.

The Los Angeles County Department of Health Services has used pulsed-field gel electrophoresis (PFGE) for outbreak investigations for 2 years. Bacteria caused 140 (24%) of 576 reported outbreaks, 36% of health facility outbreaks, but only 19% of community outbreaks. PFGE was used in 32 investigations, of which 29 (91%) were nosocomial. The most common organisms were staphylococcus and enterococcus. In contrast, PFGE was used in only 3 (4%) of 77 investigations of community bacterial outbreaks. Health departments should consider the number, setting, and causes of outbreaks that they investigate to determine if PFGE will be useful in their investigation arsenal.

The Washington State Department of Health has developed a pilot electronic laboratory-based reporting mechanism to route infectious disease reports from a large managed care organization to local health agencies. The overall goal is development of a generic electronic reporting mechanism. For this project, Health Level 7 was identified as a common ground for sharing data. Preliminary data suggest that timeliness and completeness of reporting will improve. Adhering to nationally recognized standards and codes may help reduce problems associated with transferring data; however, no public health software or implementation package was available, so resource-intensive customization was necessary.

The Maine Bureau of Health is attempting to characterize statewide hepatitis C (HCV) prevalence through a review of existing databases (hospital discharges, deaths, Medicaid registry, blood donor screening), mandatory laboratory reporting with physician questionnaire follow-up, a blinded seroprevalence survey in sexually transmitted disease clinics, and a survey of gastroenterologists. Initial data indicate dramatic increases in HCV-related hospitalization and in Medicaid expenditures during the mid-1990s and an unexpectedly high proportion of patients with injection drug–associated risk histories. Local surveillance data are useful for public policy decisions and as educational tools for physicians and the public.

